# Efficacy, safety, and tolerability of secukinumab in patients with active ankylosing spondylitis: a randomized, double-blind phase 3 study, MEASURE 3

**DOI:** 10.1186/s13075-017-1490-y

**Published:** 2017-12-22

**Authors:** Karel Pavelka, Alan Kivitz, Eva Dokoupilova, Ricardo Blanco, Marco Maradiaga, Hasan Tahir, Luminita Pricop, Mats Andersson, Aimee Readie, Brian Porter

**Affiliations:** 1 Institute of Rheumatology and Department of Rheumatology, 1st Faculty of Medicine, Charles University, Prague, Czech Republic; 2 Altoona Center for Clinical Research, Duncansville, PA USA; 3 Medical Plus, s.r.o., Uherske Hradiste, Czech Republic; 4 Division of Rheumatology, Hospital Universitario Marqués de Valdecilla, Santander, Spain; 5 Centro de Investigación de Tratamientos Innovadores de Sinaloa, Culiacán, México; 6 Barts Health NHS Trust, London, UK; 7 Novartis Pharmaceuticals Corporation, East Hanover, NJ USA; 8 Novartis Pharma AG, Basel, Switzerland

**Keywords:** Secukinumab, IL-17, Ankylosing spondylitis, Biologic therapy

## Abstract

**Background:**

Secukinumab, an anti–interleukin-17A monoclonal antibody, improved the signs and symptoms of ankylosing spondylitis (AS) in two phase 3 studies (MEASURE 1 and MEASURE 2). Here, we present 52-week results from the MEASURE 3 study assessing the efficacy and safety of secukinumab 300 and 150 mg subcutaneous maintenance dosing, following an intravenous loading regimen.

**Methods:**

A total of 226 patients were randomized to intravenous secukinumab 10 mg/kg (baseline, weeks 2 and 4) followed by subcutaneous secukinumab 300 mg (IV-300 mg) or 150 mg (IV-150 mg) every 4 weeks, or matched placebo. Patients in the placebo group were re-randomized to subcutaneous secukinumab at a dose of 300 or 150 mg at week 16. The primary endpoint was the Assessment of SpondyloArthritis international Society criteria for 20% improvement (ASAS20) response rate at week 16 in the IV-300 mg or IV-150 mg versus placebo. Other endpoints assessed through week 52 included improvements in ASAS40, ASAS 5/6, Bath Ankylosing Spondylitis Disease Activity Index, and ASAS partial remission responses, as well as the change from baseline in high-sensitivity C-reactive protein levels. Statistical analyses followed a predefined hierarchical hypothesis testing strategy to adjust for multiplicity of testing, with non-responder imputation used for binary variables and mixed-model repeated measures for continuous variables.

**Results:**

The primary efficacy endpoint was met; the ASAS20 response rate was significantly greater at week 16 in the IV-300 mg (60.5%; *P* < 0.01) and IV-150 mg (58.1%; *P* < 0.05) groups versus placebo (36.8%). All secondary endpoints were met at week 16, except ASAS partial remission in the IV-150 mg group. Improvements achieved with secukinumab in all clinical endpoints at week 16 were also sustained at week 52. Infections, including candidiasis, were more common with secukinumab than with placebo during the placebo-controlled period. During the entire treatment period, pooled incidence rates of *Candida* infections and grade 3–4 neutropenia were 1.8% for both of these adverse events in secukinumab-treated patients.

**Conclusions:**

Secukinumab (300 mg and 150 mg dose groups) provided rapid, significant and sustained improvement through 52 weeks in the signs and symptoms of patients with AS. The safety profile was consistent with previous reports, with no new or unexpected findings.

**Trial registration:**

ClinicalTrials.gov, NCT02008916. Registered on 8 December 2013. EUDRACT 2013-001090-24. Registered on 24 October 2013). The study was not retrospectively registered.

**Electronic supplementary material:**

The online version of this article (doi:10.1186/s13075-017-1490-y) contains supplementary material, which is available to authorized users.

## Background

Ankylosing spondylitis (AS) is a chronic inflammatory disease, which is mainly characterized by the involvement of the axial skeleton and the sacroiliac joints [1, 2]. AS is often associated with inflammatory back pain and stiffness, which can lead to functional impairment and reduced quality of life [3, 4].

AS affects up to 1.4% of the population worldwide and is associated with significant morbidity and disability [5, 6]. As per the treatment recommendations of the Assessment of SpondyloArthritis international Society criteria (ASAS)/European League Against Rheumatism (EULAR)/American College of Rheumatology (ACR), non-steroidal anti-inflammatory drugs (NSAIDs) are the first-line treatment for patients with active, predominantly axial manifestations of spondyloarthritis. Patients with peripheral disease who do not respond to NSAIDs may be treated with conventional disease-modifying anti-rheumatic drugs (DMARDs); nonetheless, DMARDs are not recommended in patients with axial manifestations, due to lack of efficacy [3, 7].

Although, tumor necrosis factor (TNF) inhibitors are the first-line biologic therapy for AS, up to 40% of patients do not respond to them [8, 9], collectively due to primary or secondary treatment failure or intolerance. Therefore, there remains an unmet medical need, particularly in these patients [10, 11].

The interleukin (IL)-23/IL-17 axis is known to be implicated in the pathogenic mechanism of AS [12]. Secukinumab, a fully human anti-IL-17A monoclonal antibody, has shown efficacy in the treatment of patients with AS [12–15], psoriasis [16], and psoriatic arthritis [17–19]. Previous proof-of-concept and phase 3 studies (MEASURE 1 and MEASURE 2) have shown that the inhibition of IL-17A receptor with secukinumab improves signs and symptoms in patients with AS [14, 15]. MEASURE 3 is the first phase 3 study evaluating the 300 mg subcutaneous dose of secukinumab, along with the approved 150 mg dose, in patients with moderate to severe active AS (NCT02008916).

Here, we present the efficacy and safety results of subcutaneous maintenance dosing of secukinumab 300 and 150 mg, following a 10 mg/kg intravenous loading regimen, through 52 weeks from the MEASURE 3 study.

## Methods

### Patients

Patients aged ≥ 18 years with moderate to severe AS fulfilling the Modified New York criteria for AS were enrolled in the study. Other inclusion criteria included a score of 4 or higher on the Bath Ankylosing Spondylitis Disease Activity Index (BASDAI; scores of 0–10), a spinal pain score ≥4 (out of 10) in the BASDAI item 2, and a total back pain score ≥40 mm on a 100 mm visual analog scale (VAS), despite treatment with the highest recommended dose of NSAIDs with an acceptable side effect profile for the patient.

Patients on scheduled NSAIDs were included if they had received a stable dose for at least 2 weeks before randomization. Previous use of DMARDs and anti-TNF agents was allowed. Washout periods for these agents, other than sulfasalazine and methotrexate, were required before the initiation of study treatment. Patients previously treated with not more than one anti-TNF agent could participate if they had an inadequate response to an approved dose for 3 months or more or had unacceptable side effects following at least one dose (hereafter, collectively referred to as patients with an inadequate response to anti-TNF agents). Patients could continue to receive the following medications at a stable dose: sulfasalazine (≤ 3 g per day), methotrexate (7.5–25 mg per week), prednisone or equivalent (≤ 10 mg per day), and NSAIDs.

Key exclusion criteria were total spinal ankylosis, evidence of infection or cancer on chest radiography, active systemic infection within 2 weeks before the baseline visit, and previous treatment with cell-depleting therapies or biologics other than anti-TNF agents.

### Study design

MEASURE 3 is a randomized, double-blind, double-dummy, placebo-controlled, parallel-group, 3-year study, which is being conducted at 54 centers across the USA, Belgium, Czech Republic, Germany, Greece, Mexico, Portugal, Russian Federation, Spain, and the UK. After a 10-week screening period, eligible patients were randomized (1:1:1) using an interactive response technology (IRT) system to one of two secukinumab dose groups (300 mg or 150 mg) or a placebo group. Patients in the secukinumab groups received an intravenous dose of 10 mg/kg body weight (delivered as a 125 mg/5 mL liquid-in-vial formulation) at baseline and weeks 2 and 4, followed by subcutaneous secukinumab in the form of prefilled syringes (PFS) at a dose of either 300 mg (IV-300 mg) or 150 mg (IV-150 mg) every 4 weeks starting at week 8. Patients in the placebo group were treated according to the same intravenous-to-subcutaneous administration schedule (see Additional file 1). At week 16, all patients in the placebo group were re-randomized to receive either secukinumab 300 mg or 150 mg (1:1) subcutaneously every 4 weeks.

The randomization of patients was stratified according to previous anti-TNF therapy (patients who were naïve to anti-TNF therapy (anti-TNF-naïve) or those with a history of inadequate response or intolerance to these agents (anti-TNF-IR)).

The institutional review board at each participating center approved the protocol. The trial was conducted by the study investigators in accordance with the Declaration of Helsinki and Good Clinical Practice (GCP) guidelines, and was analyzed by the Sponsor. Written informed consent was obtained from all patients. Data from the primary analysis at week 16 and the 1-year follow-up analysis (after all patients had completed the visit at week 52) are presented here.

### Outcomes

The primary endpoint was the proportion of patients who met the ASAS20 (improvement of ≥ 20% and absolute improvement of ≥ 1 unit (on a 10-unit scale) in at least three of the four main ASAS domains, with no more than 20% worsening in the remaining domain) response criteria at week 16. Secondary endpoints assessed as part of the predefined hierarchical hypothesis-testing strategy at week 16 included improvement in ASAS40 response criteria (improvement of ≥ 40% and absolute improvement of ≥ 2 units (on a 10-unit scale) in at least three of the four main ASAS domains, with no worsening in the remaining domain), change from baseline in high-sensitivity C-reactive protein (hsCRP) level, ASAS5/6 response (≥ 20% improvement in five of the six ASAS response domains), change from baseline in the total BASDAI score, and proportion of patients achieving ASAS partial remission (a score of ≤ 2 units in each of the four core ASAS domains). Primary and secondary endpoints were assessed at baseline, weeks 1, 2, 4, and every 4 weeks until week 16, thereafter all efficacy endpoints were assessed at weeks 32, 40, and 52.

PFS usability was assessed with the Self-Injection Assessment Checklist and the Possible Hazard Assessment Checklist at weeks 8 and 12. Satisfaction with the PFS was assessed with the Self-Injection Assessment Questionnaire (SIAQ) focusing on three domains: feeling about “self-injections”, “self-confidence”, and “satisfaction with self-injection”. The first part of the SIAQ (the PRE module) was completed before self-injection at baseline and the second part (the POST module) was completed after self-injection at the week 8, 12, and 16 visits.

Pre-specified subgroup analyses on the basis of anti-TNF response status (TNF-naïve vs TNF-IR) were performed for key efficacy endpoints. Safety was evaluated by means of open assessment of adverse events (AEs), serious AEs, electrocardiogram (ECG), vital signs, and routine laboratory values.

For the safety analysis during the entire treatment period (from baseline through the week 52 visit of the last patient), the secukinumab groups included any patients who received the stated dose of secukinumab, including those patients randomized to placebo at baseline who were re-randomized to active treatment at week 16. These secukinumab groups have been denoted as any secukinumab 300 mg, any secukinumab 150 mg, or any secukinumab (which includes all patients who received any dose of secukinumab, whether assigned at baseline or re-randomized to active treatment at week 16).

### Statistical analysis

Assuming an ASAS20 response rate of 60% in the secukinumab groups and 20% in the placebo group, including 74 patients in each study group, provided 99% power to reject the primary hypothesis with a 2.5% type I error rate based on Fisher’s exact test. This sample-size also provided adequate power for analysis of secondary endpoints ranging from 79% to 99%.

Primary and secondary efficacy endpoint analyses included all patients according to the treatment assigned at baseline randomization. Closed testing procedures were used to maintain a family-wise error rate of 5% across the secukinumab groups and endpoints. The hypotheses for the primary objective in either secukinumab treatment arm vs placebo were tested simultaneously at the *P* = 0.025 level. Based on the rejection of one or both of these hypotheses, secondary endpoint analysis was completed according to a pre-specified hierarchy sequence.

The primary and other binary endpoints were evaluated by means of logistic regression with treatment and anti-TNF response status as factors and weight as a covariate. Missing values, including those due to discontinuation of the study treatment, were imputed as failure to achieve the given response (non-responses).

Between-group differences in continuous variables were evaluated using the mixed-model repeated measures (MMRM) approach, with missing data assumed to be missing at random and with study group, assessment visit, and anti-TNF response status as factors. Weight and baseline values of the endpoints were included in the model as continuous covariates. Interaction terms included study group and baseline value according to the assessment visit. For changes in hsCRP, the log ratio of the post-baseline value to the baseline value was used to normalize the distribution of hsCRP at each assessment.

Safety endpoints were evaluated in the safety set, which included all patients who received at least one dose of the study drug; these endpoints were summarized descriptively. Safety results are presented for the placebo-controlled period (i.e., first 16 weeks of treatment) to reflect the time point at which the primary objective was defined and the entire safety reporting period, which included all safety data up to the date cutoff of the last patient’s week 52 clinic visit.

## Results

### Patients

**Fig. 1:**
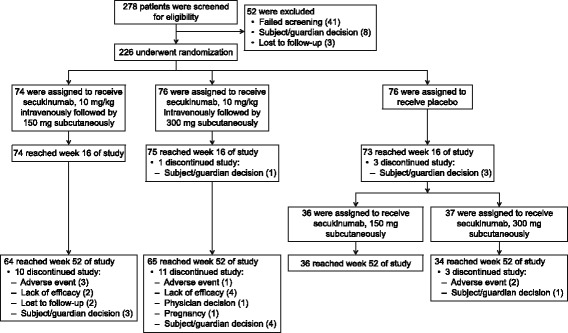
Number of patients who were screened, underwent randomization, and completed 52 weeks of the study. The secukinumab groups received intravenous secukinumab at a dose of 10 mg/kg body weight at baseline and weeks 2 and 4, followed by subcutaneous secukinumab at a dose of 300 mg or 150 mg, starting at week 8 and then every 4 weeks. The placebo group received intravenous placebo at baseline and weeks 2 and 4, followed by subcutaneous placebo every 4 weeks starting at week 8 through week 16. Patients initially assigned to receive placebo were re-randomized at week 16 to receive secukinumab 300 mg or 150 mg. Analyses of primary and secondary efficacy endpoints at week 16 included all patients according to the assigned study treatment at baseline. The most frequent reasons for screening failure included meeting the exclusion criteria of history of ongoing, chronic or recurrent infectious disease or evidence of tuberculosis infection (n = 11), not meeting the inclusion criteria of: active ankylosing spondylitis assessed by total Bath Ankylosing Spondylitis Disease Activity Index ≥4 (0–10) at baseline (n = 9) and total back pain as measured by visual analog scale ≥40 mm (0–100 mm) at baseline (n = 6)

Of 278 patients screened, 226 (81.3%) underwent randomization to receive an intravenous loading regimen of secukinumab 10 mg/kg body weight at baseline and weeks 2 and 4, followed by subcutaneous secukinumab at a dose of 300 mg (IV-300 mg; N = 76), or 150 mg (IV-150 mg; N = 74) starting from week 8. Placebo-group patients (N = 76) received an intravenous loading regimen of placebo at baseline and weeks 2 and 4, followed by subcutaneous placebo starting from week 8. Of the patients randomized, 222 (98.2%) completed the 16-week evaluation period (Fig. 1). Four patients discontinued the study before week 16 for the reasons outlined in Fig. 1. Demographics and baseline disease characteristics were similar across the study groups (Table 1). Most of the patients (97.8%) were < 65 years of age, with a median age among the groups ranging from 42.0 to 43.0 years. Approximately 24% of patients were anti-TNF-IR, two-thirds (60.2%) of the patients were male, and 72.6% were white.

**Table 1:** Demographic and baseline characteristics of the patients

Characteristic	Secukinumab IV-300 mg (*N* = 76)	Secukinumab IV-150 mg (*N* = 74)	Placebo (*N* = 76)
Age (years), mean (SD)	42.1 (11.8)	42.9 (11.1)	42.7 (11.4)
Male, *n* (%)	50 (65.8)	46 (62.2)	40 (52.6)
White, *n* (%)	52 (68.4)	54 (73.0)	58 (76.3)
Weight (kg), mean (SD)	82.7 (16.9)	80.3 (19.2)	79.0 (15.5)
Time since AS diagnosis (years), mean (SD)	5.3 (7.3)	6.0 (7.2)	5.2 (6.4)
HLA-B27-positive at baseline, *n* (%)	56 (73.7)	52 (70.3)	53 (69.7)
Anti-TNF-naïve, *n* (%)	57 (75.0)	57 (77.0)	59 (77.6)
Total BASDAI score, mean (SD)	7.0 (1.4)	7.0 (1.4)	6.9 (1.3)
hsCRP (mg/L), median (min–max)	13.3 (0.2–65.1)	21.1 (0.4–111.3)	20.0 (0.2–112.5)
Total back pain score (0–100 mm scale), mean (SD)	74.1 (15.1)	75.2 (14.9)	75 (13.9)
Baseline systemic treatment, *n* (%)			
Methotrexate use at randomization	13 (17.1)	10 (13.5)	6 (7.9)
Sulfasalazine use at randomization	20 (26.3)	14 (18.9)	19 (25.0)
Corticosteroid use at randomization	6 (7.9)	9 (12.2)	14 (18.4)
NSAID use at randomization	63 (82.9)	62 (83.8)	64 (84.2)

*n* number of patients, *SD* standard deviation, *AS* ankylosing spondylitis, *HLA* human leukocyte antigen, *TNF* tumor necrosis factor, *BASDAI* Bath Ankylosing Spondylitis Disease Activity Index, *hsCRP* high-sensitivity C-reactive protein, *NSAID* non-steroidal anti-inflammatory drugs

### Pharmacokinetics

Despite the relatively high dose of the intravenous loading regimen (10 mg/kg times three doses, based on previous pharmacokinetic and pharmacodynamics studies [20]) administered to both secukinumab groups, overall drug exposure was higher in the IV-300-mg-dose group (median concentration of 40 mcg/mL) compared to the IV-150-mg-dose group (20 mcg/mL) at week 24.

### Efficacy

**Fig. 2:**
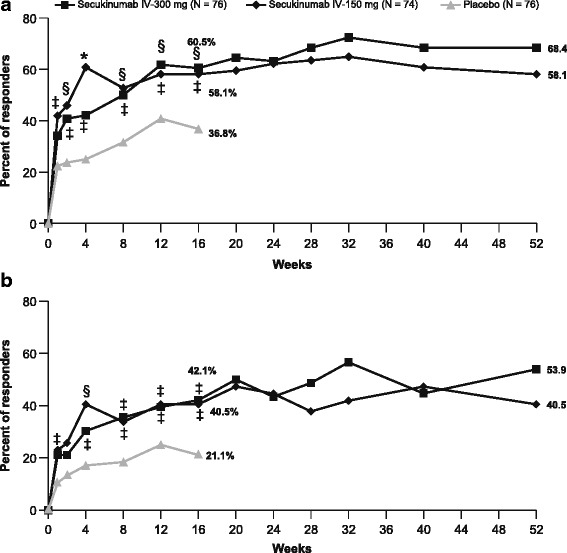
Response rates through week 16 (placebo-controlled phase) and through week 52. Shown are the proportions of patients with Assessment of SpondyloArthritis international Society 20% improvement (ASAS20) responses (improvement of ≥ 20% and absolute improvement of ≥ 1 unit (on a 10-unit scale) in at least three of the four main ASAS domains, with no worsening of ≥ 20% in the remaining domain (**a**)) and ASAS40 responses (improvement of ≥ 40% and absolute improvement of ≥ 2 units (on a 10-unit scale) in at least three of the four main ASAS domains, with no worsening in the remaining domain (**b**)). Missing data were imputed as non-responses up to week 52. *P* values at week 16 were adjusted for multiplicity of testing: ^*^ *P* < 0.0001; ^§^ *P* < 0.01; ^‡^ *P* < 0.05 versus placebo

The primary endpoint was met with both secukinumab groups at week 16; the ASAS20 response rate was 60.5% (*P* < 0.01) with secukinumab IV-300 mg and 58.1% (*P* < 0.05) with secukinumab IV-150 mg vs 36.8% with placebo (Fig. 2a), with differentiation from placebo seen as early as week 1.

Secondary endpoints were assessed in hierarchical order. ASAS40 response rates at week 16 were significant in both secukinumab groups (42.1% and 40.5% with secukinumab IV-300 and IV-150 mg, respectively) vs placebo (21.1%; *P* < 0.05 for both comparisons vs placebo; Fig. 2b). All other predefined secondary endpoints were also met in both secukinumab groups, except ASAS partial remission in the secukinumab IV-150 mg group. Significantly greater mean decreases from baseline in hsCRP (represented by lower post-baseline to baseline ratios) were observed with both secukinumab groups vs placebo (0.48 for secukinumab IV-300 mg and 0.55 for secukinumab IV-150 mg versus 1.09 for placebo; *P* < 0.05 for both secukinumab groups vs placebo). Both secukinumab groups achieved significantly higher ASAS 5/6 response rates compared with the placebo group at week 16 (39.5% for secukinumab IV-300 mg and 41.9% for secukinumab IV-150 mg vs 14.5% for placebo; *P* < 0.05 for both secukinumab groups vs placebo). The improvement in total BASDAI score from baseline to week 16 was also significantly greater for both secukinumab groups compared with the placebo group (−2.69 for secukinumab IV-300 mg and −2.28 for secukinumab IV-150 mg versus −1.45 for placebo; *P* < 0.05 for both secukinumab groups versus placebo). The proportion of patients achieving ASAS partial remission at week 16 was significantly higher in the secukinumab IV-300 mg group (21.1%; *P* < 0.05) compared with the placebo group (1.3%), while the secukinumab IV-150 mg group (9.5%) did not significantly differ from placebo on the basis of hierarchical testing (Table 2).

**Table 2:** Efficacy endpoints at week 16 using non-responder imputation

Endpoints	Secukinumab IV-300 mg (*N* = 76)	Secukinumab IV-150 mg (*N* = 74)	Placebo (*N* = 76)
ASAS20, *n* (%)	46 (60.5)^§^	43 (58.1)^‡^	28 (36.8)
ASAS40, *n* (%)	32 (42.1)^‡^	30 (40.5)^‡^	16 (21.1)
hsCRP (post-baseline/baseline ratio), mean change from baseline ± SE	0.48 ± 1.1^‡^	0.55 ± 1.1^‡^	1.09 ± 1.1
ASAS 5/6, *n* (%)	30 (39.5)^‡^	31 (41.9)^‡^	11 (14.5)
BASDAI, mean change from baseline ± SE	-2.7 ± 0.3^‡^	-2.3 ± 0.3^‡^	-1.5 ± 0.3
ASAS partial remission, *n* (%)	16 (21.1)^‡^	7 (9.5)	1 (1.3)

Non-responder imputation (binary variables) and mixed-model repeated measures (continuous variables) data are presented

*ASAS20* 20% response according to criteria of the Assessment of Spondyloarthritis International Society, *ASAS40* 40% response according to ASAS criteria, *hsCRP* high-sensitivity C-reactive protein, *BASDAI* Bath Ankylosing Spondylitis Disease Activity Index

*ASAS* Assessment of SpondyloArthritis international Society, *BASDAI* Bath Ankylosing Spondylitis Disease Activity Index, *hsCRP* high-sensitivity C-reactive protein, *N* number of patients, *SE* standard error

^‡^ *P* < 0.05, ^§^ *P* < 0.01 vs placebo. *P* values were adjusted for multiplicity of testing

In pre-specified subgroup analyses at week 16, ASAS20 responses were higher in both secukinumab groups vs placebo, regardless of anti-TNF status. ASAS20 response rates in anti-TNF-naïve patients were 64.9% and 63.2% in the secukinumab IV-300 and IV-150 mg groups, respectively, vs 39.0% with placebo; the corresponding rates in TNF-IR patients were 47.4% and 41.2% vs 29.4%, respectively. In these subgroup analyses, numerical improvements were also observed in both secukinumab groups vs placebo in the secondary efficacy endpoints (ASAS40, hsCRP, ASAS5/6, BASDAI, and ASAS partial remission), regardless of anti-TNF status (Table 3).

**Table 3:** Efficacy endpoints at week 16 by anti-TNF status

Endpoints	Secukinumab IV-300 mg	Secukinumab IV-150 mg	Placebo
Anti-TNF-naïve	*N* = 57	*N* = 57	*N* = 59
ASAS20, *n* (%)	37 (64.9)^§^	36 (63.2)^‡^	23 (39.0)
ASAS40, *n* (%)	25 (43.9)^‡^	25 (43.9)^‡^	14 (23.7)
hsCRP (post-baseline/baseline ratio), LS mean change ± SE	0.43 ± 1.1^*^	0.51 ± 1.1^†^	0.91 ± 1.1
ASAS 5/6, *n* (%)	24 (42.1)^‡^	26 (45.6)^§^	11 (18.6)
BASDAI, LS mean change ± SE	-3.2 ± 0.3^§^	-2.6 ± 0.3	-1.9 ± 0.3
ASAS partial remission, *n* (%)	12 (21.1)^§^	6 (10.5)	1 (1.7)
Anti-TNF-IR	*N* = 19	*N* = 17	*N* = 17
ASAS20, *n* (%)	9 (47.4)	7 (41.2)	5 (29.4)
ASAS40, *n* (%)	7 (36.8)	5 (29.4)	2 (11.8)
hsCRP (post-baseline/baseline ratio), LS mean change ± SE	0.57 ± 1.2^§^	0.58 ± 1.3^§^	1.5 ± 1.2
ASAS 5/6, *n* (%)	6 (31.6)^‡^	5 (29.4)^‡^	0
BASDAI, LS mean change ± SE	-1.8 ± 0.6	-2.2 ± 0.6	-0.9 ± 0.6
ASAS partial remission, *n* (%)	4 (21.1)	1 (5.9)	0

Non-responder imputation (binary variables) and mixed-model repeated measures (continuous variables) analyses are presented

*TNF* tumor necrosis factor, *N* number of patients, *ASAS* Assessment of SpondyloArthritis international Society, *hsCRP* high-sensitivity C-reactive protein, *LS* least squares, *SE* standard error, *BASDAI* Bath Ankylosing Spondylitis Disease Activity Index

^‡^ *P* < 0.05, ^§^ *P* < 0.01, ^†^ *P* < 0.001, ^*^ *P* < 0.0001 vs placebo. *P*-values are unadjusted

### Long-term efficacy

A total of 199 (88.1%) patients completed the 52-week evaluation period; 27 patients discontinued the study before week 52 due to the reasons depicted in Fig. 1. ASAS20 and ASAS40 response rates observed at week 16 in both secukinumab groups were sustained through 52 weeks of therapy, on the basis of both observed data and a more conservative estimate of efficacy with missing values imputed as non-response (Fig. 2 and Additional file 2). At week 52, ASAS20 and ASAS40 response rates using non-responder imputations were 68.4% and 53.9%, and 58.1% and 40.5% with secukinumab IV-300 mg and IV-150 mg, respectively. Improvements achieved with secukinumab in all other secondary endpoints at week 16 were also sustained at week 52 (see Additional file 2). Improvements in ASAS20 and ASAS40 response rates, and all other endpoints were observed in placebo-treated patients re-randomized at week 16 to either secukinumab 300 or 150 mg through week 52 (see Additional file 3).

Consistent with the results on usability and satisfaction with the PFS from previous secukinumab studies in patients with psoriatic arthritis and psoriasis [21], all patients reported successful self-administration of study treatment using the PFS at week 12. “Needle stick in a non-critical area” was the only hazard experienced across all treatment groups (total 4.6%). Overall, SIAQ domain scores improved over time across all treatment groups (see Additional file 4).

### Safety

#### The 16-week placebo-controlled period

During the 16-week placebo-controlled period, AEs were reported in 44.7% of patients in the secukinumab IV-300 mg group, 45.9% of patients in the secukinumab IV-150 mg group, and 44.0% of patients in the placebo group. The most frequent treatment-emergent AEs during week 16 were nasopharyngitis, diarrhea, headache, and cough. Through week 16, none of the patients discontinued treatment due to an AE. Rates of non-fatal SAEs and discontinuations were low and similar across the study groups (Table 4). Treatment-emergent SAEs were reported in one patient each in the secukinumab IV-300 mg group (ankylosing spondylitis flare) and placebo group (non-serious urinary tract infection), neither of which led to interruption of study treatment.

**Table 4:** Safety profile during week 16 (placebo-controlled period) and the entire safety reporting period

Variable	Through week 16 (placebo-controlled period)^a^			Entire safety-data period		
	Secukinumab IV-300 mg (*N* = 76)	Secukinumab IV-150 mg (*N* = 74)	Placebo (*N* = 75)	Any secukinumab 300 mg (*N* = 113)	Any secukinumab 150 mg (*N* = 110)	Any secukinumab (*N* = 223)
Exposure to study treatment, days, mean **±** SD	112.9 **±** 14.2	117.4 ± 13.1	112.2 ± 19.4	410.7 ± 108.9	425.9 ± 92.4	418.2 ± 101.1
	Number of patients (%)			Number of patients (incidence rate per 100 patient years)		
Any AE	34 (44.7)	34 (45.9)	33 (44.0)	83 (152.7)	90 (179.2)	173 (165.4)
Serious AE	1 (1.3)	0 (0.0)	1 (1.3)	6 (4.8)	6 (4.8)	12 (4.8)
Discontinued due to AEs	0 (0.0)	0 (0.0)	0 (0.0)	4 (3.5)	4 (3.6)	8 (3.6)
Death	0 (0.0)	0 (0.0)	0 (0.0)	0 (0.0)	0 (0.0)	0 (0.0)
Common AEs^a^						
Nasopharyngitis	3 (3.9)	6 (8.1)	2 (2.7)	16 (13.7)	22 (19.6)	38 (16.6)
Diarrhea	3 (3.9)	5 (6.8)	0 (0.0)	8 (6.6)	9 (7.5)	17 (7.0)
Headache	3 (3.9)	5 (6.8)	5 (6.7)	11 (9.1)	12 (10.1)	23 (9.6)
Cough	3 (3.9)	3 (4.1)	1 (1.3)	5 (4.1)	3 (2.4)	8 (3.2)
Pharyngitis	3 (3.9)	1 (1.4)	1 (1.3)	4 (3.2)	3 (2.4)	7 (2.8)
Ear infection	3 (3.9)	0 (0.0)	0 (0.0)	3 (2.4)	0	3 (1.2)
Urinary tract infection	3 (3.9)	0 (0.0)	3 (4.0)	6 (4.9)	3 (2.4)	9 (3..6)
AEs of special interest						
*Candida* infection	1 (1.4)	1 (1.3)	0 (0.0)	2 (1.8)	2 (1.8)	4 (1.8)
Malignant tumor	0 (0.0)	0 (0.0)	0 (0.0)	1 (0.9)	1 (0.9)	2 (0.9)
Neutropenia						
Grade 3	0 (0.0)	1 (1.4)	0 (0.0)	1 (0.9)	1 (0.9)	2 (0.9)
Grade 4	0 (0.0)	2 (2.7)	0 (0.0)	0 (0.0)	2 (1.8)	2 (0.9)

The entire safety reporting period includes all safety data up to the date cutoff of the last patient’s week 52 clinic visit. One patient was excluded from placebo group as no treatment was given after randomization. In the analysis of the entire study period, the secukinumab groups include any patients who received the stated dose of secukinumab, including those who were randomly assigned to the placebo group at baseline and who underwent a second randomization to active treatment at week 16

*N* number of patients, *AE* adverse event, *SD* standard deviation

^a^AEs with frequency ≥3% in either of the two secukinumab groups during the 16-week placebo-controlled period

### Entire safety reporting period

During the entire safety reporting period, the maximum duration of exposure to secukinumab in the “any secukinumab” group was 607 days, with a median exposure of 428.0 days. The exposure-adjusted incidence rate of non-fatal SAEs was low and was similar between both secukinumab groups (4.8 events per 100 patient-years in the any secukinumab 300 mg and 150 mg groups).

No cases of adjudicated major adverse cardiovascular events, Crohn’s disease, or ulcerative colitis were reported. Two patients (one case each in the any secukinumab 150 mg and any secukinumab 300 mg) group reported grade 3 neutropenia; grade 4 neutropenia was reported in two patients from the any secukinumab 150 mg group. These findings resolved, did not lead to discontinuation of study treatment, and no concomitant infections or infestations were reported. *Candida* infection was reported in two patients each in the any secukinumab 300 mg and 150 mg dose groups. These included one case each of esophageal candidiasis, vulvovaginal candidiasis, genital candidiasis and oral candidiasis. All these cases were mild to moderate in severity and resolved with standard antifungal treatment, without leading to study discontinuation. Uveitis was reported in one patient in the any secukinumab 300 mg group and in two patients in the any secukinumab 150 mg group (with one new-onset mild event leading to treatment discontinuation, while the other two cases were in patients with a prior history of uveitis). Benign tumor was reported in one patient each in the any secukinumab 300 mg (skin papilloma) and 150 mg (enchondroma) groups.

No other clinically relevant changes were observed in laboratory parameters, vital signs, and ECG findings over the entire safety reporting period. No dose dependence was observed in any safety risk. Treatment-emergent anti-drug antibodies were reported in only one patient in the secukinumab IV-300 mg group, which were not neutralizing to secukinumab and did not lead to loss of efficacy, pharmacokinetic abnormalities, or associated immunogenicity-related AEs. No deaths were reported during the study.

## Discussion

Both secukinumab groups (300 mg and 150 mg subcutaneous maintenance dosing, preceded by an intravenous loading regimen) had rapidly reduced signs and symptoms of active AS, further confirming the results from two previous phase 3 studies that used either intravenous or subcutaneous loading [15]. Placebo-treated patients re-randomized to secukinumab at week 16 (no loading regimen) also showed improvements across all efficacy endpoints through 52 weeks of treatment.

As in previous studies, the current trial met the primary endpoint with approximately 60% of patients achieving an ASAS20 response in each secukinumab group, with an early onset of action by week 1 and sustained treatment responses through week 52 [15]. The bulk of the data collection in MEASURE 3 occurred following regulatory approval of secukinumab for the treatment of AS, which could potentially have contributed to increased placebo responses on patient-reported outcome measures (due to patient expectations). If this was the case, changes in objective markers of inflammation, such as hsCRP levels, would not be subject to this same placebo response, which was confirmed by the lack of decrease in hsCRP in the placebo group.

Improvements observed in more stringent ASAS40 and ASAS5/6 response criteria were more than 40% across both secukinumab groups up to 52 weeks of treatment, which provides further evidence of clinically meaningful long-term improvements in AS with secukinumab. Although MEASURE 3 is the first study to assess the efficacy and safety of a 300 mg dose of secukinumab in AS, the intravenous loading regimen used in both the 300 mg and 150 mg dose groups may have obscured dose-dependent differences in efficacy at week 16, although dose-dependent drug exposure was observed by week 24, with the IV-300 mg dose group demonstrating a median secukinumab concentration that was double that in the IV-150 mg group by this time point. Some differences in efficacy responses were evident by week 52, particularly for higher-hurdle efficacy endpoints such as ASAS40 (IV-300 mg, 53.9%; IV-150 mg, 40.5%) and ASAS partial remission (IV-300 mg, 22.4%; IV-150 mg, 16.2%).

Although TNF inhibitors have shown efficacy in patients with AS, an unmet medical need persists due to primary and secondary treatment failure and intolerance, which has been well-documented in a substantial proportion of patients with AS [10, 22]. Addressing this need, secukinumab has demonstrated efficacy in patients with AS with an inadequate response or intolerance to anti-TNF therapy in this and previous studies in AS [15]. In addition, in patients who were naïve to anti-TNF therapy for whom secukinumab was their first biologic treatment, ASAS20 response rates were approximately 5% higher with secukinumab IV-300 mg and IV-150 mg after 16 weeks of treatment, compared to the overall study population.

The safety profile of secukinumab in this study was consistent with that observed in previous trials of secukinumab in patients with active AS [14, 15] and other indications, including psoriatic arthritis [17, 18] and psoriasis [16]. No new or unexpected safety findings were reported in this study, with lower AE and SAE rates observed than previously reported in the MEASURE 1 or MEASURE 2 studies, despite the use of a higher secukinumab dose (300 mg subcutaneous monthly dosing following three weight-based 10 mg/kg intravenous loading doses), suggesting that secukinumab 300 mg is as well-tolerated as the currently approved 150 mg dose.

The use of immunomodulatory biologics has been associated with an elevated risk of AEs including *Candida* infections [23], uveitis [24], malignancy [25] and neutropenia [24, 26]. TNF inhibitors, in particular, have been associated with an increased risk of serious infections, including reactivation tuberculosis, bacterial sepsis, invasive fungal infections (such as histoplasmosis), and infections due to opportunistic pathogens [27, 28]. In the current study, for the any secukinumab 300-mg and 150-mg dose groups, the exposure-adjusted incidence rates for serious infections were 1.6 and 0.8 per 100 patient-years, respectively, over the entire safety reporting period. Absolute rates of serious infection were also comparable to placebo (secukinumab IV-300 mg, 3.9%; secukinumab IV-150 mg, 5.4%; placebo, 8%) over the first 16 weeks of treatment (placebo-controlled period). Rates of other AEs of clinical interest were similarly reassuring and comparable to placebo, including *Candida* infection, uveitis, malignancy, and grade 3 or 4 neutropenia, with no cases of Crohn’s disease or ulcerative colitis reported.

## Conclusions

In conclusion, this study reinforces the observation that secukinumab provides rapid, significant, and sustained improvement over 52 weeks in the signs and symptoms of active AS, regardless of prior experience with TNF inhibitors, with response rates that were highest in patients naïve to anti-TNF therapy. Also, the usability of the PFS and acceptability of a liquid-in-vial formulation to deliver intravenous loading doses was confirmed. These 52-week results demonstrate that both the 300 mg and 150 mg doses of secukinumab are efficacious and well-tolerated.

## Additional files

Additional file 1: Study design. Randomization was stratified according to whether patients were anti-TNF-naïve or had previous inadequate response or intolerance to anti-TNF therapy. ASAS, Assessment of SpondyloArthritis international Society; ASAS20, 20% improvement in ASAS criteria; BL, baseline; i.v., intravenous; q4wk, every 4 weeks; PBO, placebo; R, randomization; s.c., subcutaneous; TNF, tumor necrosis factor; wk, week. (PDF 817 kb)

Additional file 2: Efficacy endpoints at week 52 using non-responder imputation and observed data. (DOCX 16 kb)

Additional file 3: Efficacy endpoints at week 52 for placebo patients re-randomized to secukinumab using non-responder imputation and observed data. (DOCX 16 kb)

Additional file 4: SIOQ domain scores over week 16. Overall patient experience with secukinumab administration via the pre-filled syringes was assessed over time from baseline (PRE module) to week 16 (POST modules) by SIAQ domains: (a) feeling about self-injection; (b) self-confidence and (c) satisfaction with self-injection. SIAQ, Self-Injection Assessment Questionnaire. (PDF 781 kb)

Additional file 5: List of Independent Ethics Committees (IECs) or Institutional Review Boards (IRBs) that approved study. (DOCX 19 kb)

Additional file 6: List of ethical approval reference numbers for each participating center of this study. (DOCX 23 kb)

## Abbreviations

ACR: American College of Rheumatology
AE: Adverse event
AS: Ankylosing spondylitis
ASAS: Assessment of SpondyloArthritis international Society criteria
BASDAI: Bath Ankylosing Spondylitis Disease Activity Index
DMARDs: Disease-modifying anti-rheumatic drugs
ECG: Electrocardiogram
EULAR: European League Against Rheumatism
GCP: Good Clinical Practice
hsCRP: High sensitivity C-reactive protein
IFU: Instructions for use
IL: Interleukin
IRT: Interactive response technology
NSAID: Non-steroidal anti-inflammatory drug
PFS: Pre-filled syringe
SAE: Serious adverse event
SIAQ: Self-Injection Assessment Questionnaire
TNF: Tumor necrosis factor
TNF-IR: Tumor necrosis factor inhibitor inadequate responder
VAS: Visual analog scale
