# Supplementary figures and images for: Efficacy, safety, and tolerability of secukinumab in patients with active ankylosing spondylitis: a randomized, double-blind phase 3 study, MEASURE 3
Source: Arthritis Res Ther. 2017 Dec 22;19:285. doi: 10.1186/s13075-017-1490-y (PMC5741872; doi:10.1186/s13075-017-1490-y)

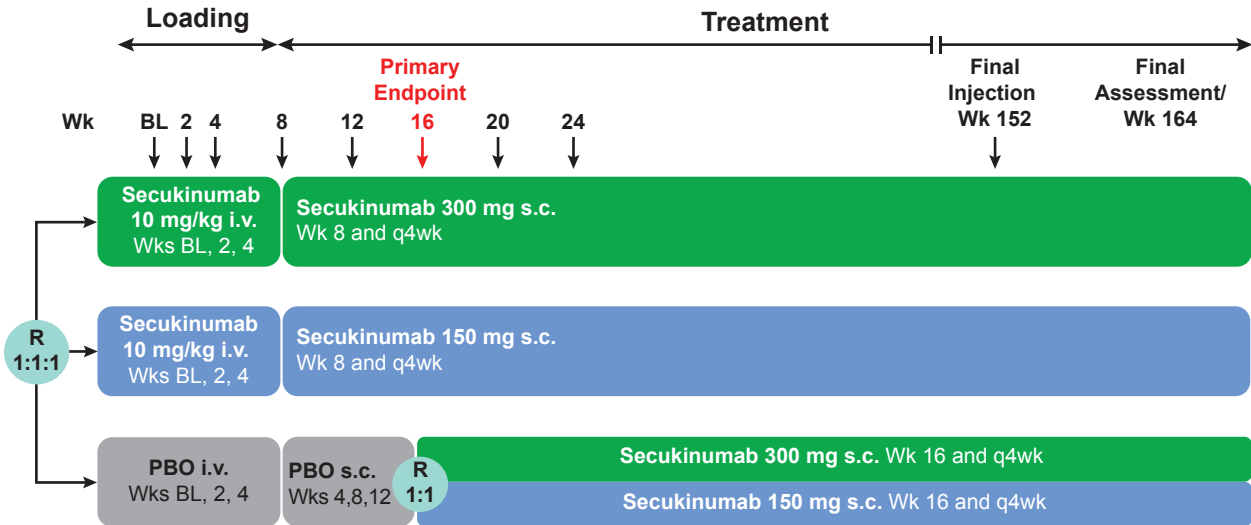

Supplement: Supplementary file 1 — Study design. Randomization was stratified according to whether patients were anti-TNF-naïve or had previous inadequate response or intolerance to anti-TNF therapy. ASAS, Assessment of SpondyloArthritis international Society; ASAS20, 20% improvement in ASAS criteria; BL, baseline; i.v., intravenous; q4wk, every 4 weeks; PBO, placebo; R, randomization; s.c., subcutaneous; TNF, tumor necrosis factor; wk, week. (PDF 817 kb) [file 13075_2017_1490_MOESM1_ESM.pdf]

■ Feeling about injections

● Self-confidence

◆ Satisfaction with self-injection

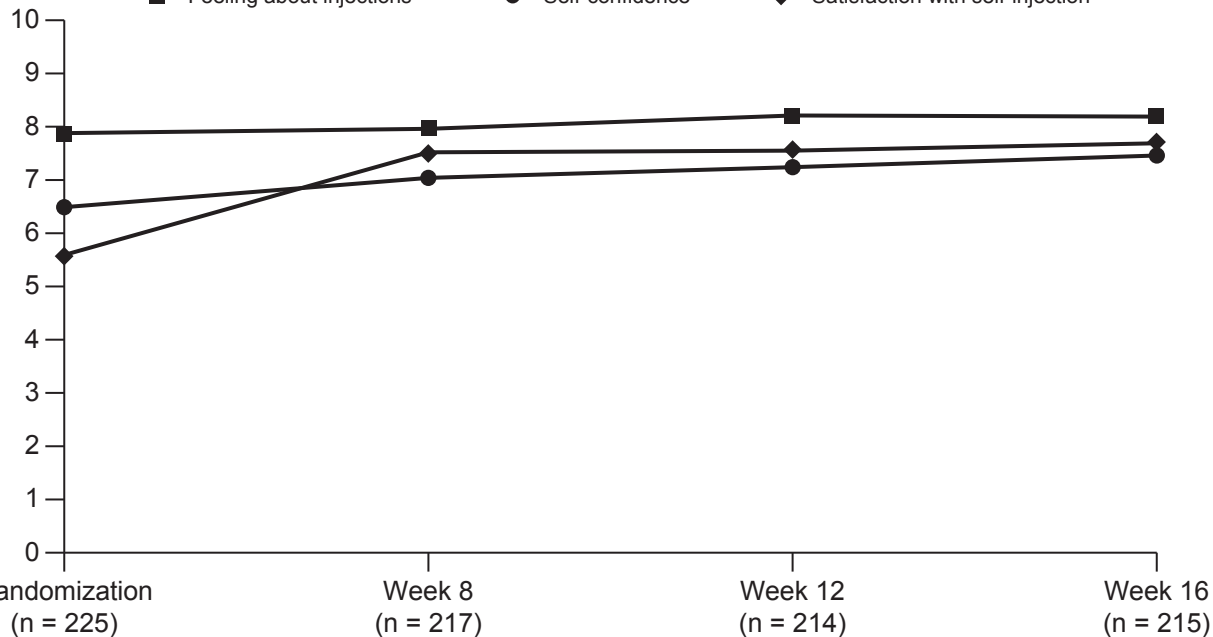

Supplement: Supplementary file 4 — SIOQ domain scores over week 16. Overall patient experience with secukinumab administration via the pre-filled syringes was assessed over time from baseline (PRE module) to week 16 (POST modules) by SIAQ domains: (a) feeling about self-injection; (b) self-confidence and (c) satisfaction with self-injection. SIAQ, Self-Injection Assessment Questionnaire. (PDF 781 kb) [file 13075_2017_1490_MOESM4_ESM.pdf]
