# Supplementary material for: Efficacy, safety, and tolerability of secukinumab in patients with active ankylosing spondylitis: a randomized, double-blind phase 3 study, MEASURE 3
Source: Arthritis Res Ther. 2017 Dec 22;19:285. doi: 10.1186/s13075-017-1490-y (PMC5741872; doi:10.1186/s13075-017-1490-y)
Supplement: Supplementary file 2 — Efficacy endpoints at week 52 using non-responder imputation and observed data. (DOCX 16 kb) [file 13075_2017_1490_MOESM2_ESM.docx]

**Efficacy endpoints at week 52 using non-responder imputation and observed data**

|  | **Imputed** | | **Observed** | |
| --- | --- | --- | --- | --- |
| **Endpoints** | **Secukinumab  IV-300 mg**  **(N = 76)** | **Secukinumab  IV-150 mg**  **(N = 74)** | **Secukinumab  IV-300 mg**  **(N = 65)** | **Secukinumab  IV-150 mg**  **(N = 59)** |
| **ASAS20, n/M (%)** | 52/76 (68.4) | 43/74 (58.1) | 52/65 (80.0) | 43/59 (72.9) |
| **ASAS40, n/M (%)** | 41/76 (53.9) | 30/74 (40.5) | 41/65 (63.1) | 30/59 (50.8) |
| **hsCRP (post-baseline/baseline ratio), mean change from baseline ± SD (observed) or SE (imputed)** | 0.5 **±** 1.1 | 0.6 **±**1.1 | -7.2 ± 12.8 | -9.4 ± 18.3 |
| **ASAS 5/6, n/M (%)** | 40/76 (52.6) | 29/74 (39.2) | 40/65 (61.5) | 29/59 (49.2) |
| **BASDAI, mean change from baseline ± SD (observed) or SE (imputed)** | -3.0 **±** 0.3 | -2.8 **±** 0.3 | -3.6 **±** 2.5 | -3.2 **±** 2.3 |
| **ASAS partial remission, n/M (%)** | 17/76 (22.4) | 12/74 (16.2) | 17/65 (26.2) | 12/59 (20.3) |
| For continuous variables, mean change from baseline is reported for observed data and least-square mean change where mixed model repeated measures analysis was performed.  ASAS denotes Assessment of SpondyloArthritis International Society criteria; BASDAI, Bath Ankylosing Spondylitis Disease Activity Index; hsCRP, high-sensitivity C-reactive protein; SD, standard deviation; SE, standard error; M, number of evaluable patients; N, number of patients randomized; n, number of patients with response. | | | | |
