# Supplementary material for: Efficacy, safety, and tolerability of secukinumab in patients with active ankylosing spondylitis: a randomized, double-blind phase 3 study, MEASURE 3
Source: Arthritis Res Ther. 2017 Dec 22;19:285. doi: 10.1186/s13075-017-1490-y (PMC5741872; doi:10.1186/s13075-017-1490-y)
Supplement: Supplementary file 3 — Efficacy endpoints at week 52 for placebo patients re-randomized to secukinumab using non-responder imputation and observed data. (DOCX 16 kb) [file 13075_2017_1490_MOESM3_ESM.docx]

**Efficacy endpoints at week 52 for placebo patients re-randomized to secukinumab using non-responder imputation and observed data**

|  | **Imputed** | | **Observed** | |
| --- | --- | --- | --- | --- |
| **Endpoints** | **Placebo-Secukinumab  300 mg**  **(N = 37)** | **Placebo-Secukinumab  150 mg**  **(N = 36)** | **Placebo-Secukinumab  300 mg**  **(N = 37)** | **Placebo-Secukinumab  150 mg**  **(N = 36)** |
| **ASAS20, n/M (%)** | 20/37 (54.1) | 23/36 (63.9) | 20/31 (64.5) | 23/36 (63.9) |
| **ASAS40, n/M (%)** | 14/37 (37.8) | 15/36 (41.7) | 14/31 (45.2) | 15/36 (41.7) |
| **hsCRP (post-baseline/baseline ratio), mean change from baseline ± SD (observed) or SE (imputed)** | 0.6 **±** 1.2 | 0.6 **±** 1.2 | -5.2 **±** 12.5 | -11.3 **±** 18.0 |
| **ASAS 5/6, n/M (%)** | 15/37 (40.5) | 17/36 (47.2) | 15/31 (48.4) | 17/36 (47.2) |
| **BASDAI, mean change from baseline ± SD (observed) or SE (imputed)** | -2.8 **±** 0.4 | -2.5 **±** 0.4 | -2.9 **±** 2.2 | -2.8 **±** 2.0 |
| **ASAS partial remission, n/M (%)** | 7/37 (18.9) | 3/36 (8.3) | 7/31 (22.6) | 3/36 (8.3) |
| For continuous variables, mean change from baseline is reported for observed data and least-square mean change where mixed model repeated measures analysis was performed.  ASAS denotes Assessment of SpondyloArthritis International Society criteria; BASDAI, Bath Ankylosing Spondylitis Disease Activity Index; hsCRP, high-sensitivity C-reactive protein; SD, standard deviation; SE, standard error; M, number of evaluable patients; N, number of patients randomized; n, number of patients with response. | | | | |
