# Supplementary material for: Efficacy, safety, and tolerability of secukinumab in patients with active ankylosing spondylitis: a randomized, double-blind phase 3 study, MEASURE 3
Source: Arthritis Res Ther. 2017 Dec 22;19:285. doi: 10.1186/s13075-017-1490-y (PMC5741872; doi:10.1186/s13075-017-1490-y)
Supplement: Supplementary file 5 — List of Independent Ethics Committees (IECs) or Institutional Review Boards (IRBs) that approved study. (DOCX 19 kb) [file 13075_2017_1490_MOESM5_ESM.docx]

**List of Independent Ethics Committees (IECs) or Institutional Review Boards (IRBs) that approved study**

| **Ethics Committee or**  **Institutional Review Board** | **Department / Organization** | **City, State/Province, Postal**  **Code Country** |
| --- | --- | --- |
| Etika komise Revmatologickeho  ustavu | Ethics Committee | Praha 2 Czech Republic128 50  Czech Republic |
| Eticka komise Pri Institutu Klinicke a  Experimentalni Mediciny a  Thomayerove Nemocnici s  Multicentrickou Pusobnost | eticka komise | Praha 4 Czech Republic 140 59  Czech Republic |
| Comité d'Ethique Hôpital Erasme | Ethics Committee | Bruxelles Belgium  Belgium |
| Ethisch Comité OLV Aalst |  | Aalst  Belgium |
| Landesamt für Gesundheit und  Soziales  Ethik-Kommission des Landes Berlin |  | Berlin  Germany |
| Friedrich-Alexander-Universität  Erlangen-Nürnberg  Medizinische Fakultät  Ethik-Kommission |  | Erlangen  Germany |
| Universitätsklinikum Jena  Ethik-Kommission |  | Jena 07740  Germany |
| Ethik-Kommission der Bayerischen  Landesärztekammer |  | München  Germany |
| Universität Göttingen  Medizinische Fakultät  Ethik-Kommission |  | Göttingen  Germany |
| Ethik-Kommission der Medizinischen  Hochschule Hannover |  | Hannover  Germany |
| Ethik-Kommission der Bayerischen  Landesärztekammer |  | München  Germany |
| Landesamt für Verbraucherschutz Ethik-Kommission des Landes Sachsen-Anhalt |  | Dessau-Roßlau 06846  Germany |
| Landesärztekammer Brandenburg Ethik-Kommission |  | Cottbus 03044  Germany |
| Ärztekammer Hamburg Geschäftsstelle der Ethik- Kommission |  | Hamburg  Germany |
| Ethikkommission der Ärztekammer  Westfalen-Lippe und der Med. Fakultät der Westfälischen Wilhelms-Universität Münster |  | Münster  Germany |
| Universität Rostock Medizinische Fakultät Ethikkommission |  | Rostock Germany |
| National Ethics Committee |  | Cholargos 15562 Greece |
| Comité Bioéthico para la Investigación Clinica |  | Mexico DF México |
| Quorum Review IRB |  | Seattle WA USA |
| Comissão de Ética para a Investigacão Clínica | Ministério de Saúde | Lisboa 1749-004 Portugal |
| LEC of medical clinic "Vashe Zdorovie" |  | Kazan Russia |
| LEC of State Medical University |  | Saint-Petersburg |
| LEC of City Hospital #25 |  | Saint-Petersburg  Russia |
| LEC of Rheumatology Inst. of  Russian Academy of Medical  Sciences |  | Saint-Petersburg  Russia |
| Secretaría Técnica del Comité Ético  de Investigación Clínica | HOSPITAL  UNIVERSITARIO LA PAZ | Madrid Madrid  Spain |
| Comité Ético de Investigación Clínica | Instituto de Formación e  Investigación Marqués de  Valdecilla | Santander Santander  Spain |
| Comité Ético de Investigación Clínica | HOSPITAL  UNIVERSITARIO REINA  SOFÍA  Edif. Consultas Externas,  nivel -1 | Córdoba Córdoba  Spain |
| Comité Ético de Investigación Clínica  de Galicia | Subdirección de Farmacia y  Productos Sanitarios  Conselleria de Sanidad | Santiago de Compostela  Spain |
| CEIC Corporació Sanitària Parc  Taulí | Fundació Parc Taulí  Edificio Santa Fe  Ala izquierda, 2ª Planta | Sabadell Barcelona  Spain |
| NRES Committee East Midlands -  Northampton | Royal Standard Place | Nottingham NG1 6FS  UK |
