# Supplementary material for: Efficacy, safety, and tolerability of secukinumab in patients with active ankylosing spondylitis: a randomized, double-blind phase 3 study, MEASURE 3
Source: Arthritis Res Ther. 2017 Dec 22;19:285. doi: 10.1186/s13075-017-1490-y (PMC5741872; doi:10.1186/s13075-017-1490-y)
Supplement: Supplementary file 6 — List of ethical approval reference numbers for each participating center of this study. (DOCX 23 kb) [file 13075_2017_1490_MOESM6_ESM.docx]

**List of Ethical Approval Reference Numbers for Each Participating Center of This Study**

| **Site Numbers** | **Centre Name** | **City, Country** | **Investigator Name** | **Institutional Review Board Reference Numbers** |
| --- | --- | --- | --- | --- |
| 1000 | Revmatologicky ustav | Praha 2, Czech Republic | Karel Pavelka | 5637/2013 |
| 1001 | Medical plus s r o | Uherske Hradiste, Czech Republic | Eva Dokoupilova | 2102/13 |
| 1051 | Hospital Erasme | Bruxelles, Belgium | Joelle Margaux | P2014/008 |
| 1053 | Onze Lieve Vrouwziekenhuis Aalst | Aalst, Belgium | Bert Vander Cruyssen | 2014/001 |
| 2001 | Charite Berlin Campus Benjamin Franklin | Berlin, Germany | Joachim Sieper | 1990 |
| 2003 | Immanuel Krankenhaus Berlin Buch | Berlin, Germany | Andreas Krause | 1990 |
| 2004 | Universitätsklinikum Erlangen-Nürnberg | Erlangen, Germany | Axel Hueber | 1990 |
| 2006 | Friedrich Schiller Universitaet Jena | Jena, Germany | Peter Oelzner | 1990 |
| 2008 | Praxiszentrum St. Bonifatius | München, Germany | Klaus Krueger | 1990 |
| 2009 | Universitätsklinikum Göttingen | Göttingen, Germany | Gerhard A.Mueller | 1990 |
| 2010 | Medizinische Hochschule Hannover | Hannover, Germany | Reinhold E. Schmidt | 1990 |
| 2011 | Rheumatologische Schwerpunktpraxis Erlangen | Erlangen, Germany | Florian Schuch | 1990 |
| 2012 | ZeFor GmbH Dr. Spieler | Zerbst, Germany | Wolfgang Spieler | 1990 |
| 2014 | Rheuma Research Lausitz Praxis Dr. med. Sutowicz | Cottbus, Germany | Mario Sutowicz | 1990 |
| 2017 | Klinikum Eilbek - Schoen Kliniken | Hamburg, Germany | Juergen Wollenhaupt | 1990 |
| 2020 | Rheumazentrum Ruhrgebiet St Josefs Krankenhaus | Herne, Germany | Juergen Braun | 1990 |
| 2021 | Institut fuer Praeventive Medizin & Klinische Forschung GbR | Magdeburg, Germany | Ruediger Moericke | 1990 |
| 2022 | Rheumazentrum Bad Doberan | Bad Doberan, Germany | Gunther Neeck | 1990 |
| 3000 | Gen. Hosp. of Athens Ippokratio | Athens, Greece | Dimitrios Vassilopoulos | 19059 |
| 3001 | General Hospital of Athens Laiko | Athens, Greece | Petros Sfikakis | ΓΣ1934 |
| 3002 | General Hospital of Heraklion Crete | Heraklion, Greece | Prodromos Sidiropoulos | 15921 |
| 3003 | 424 General Military Hospital of Thessaloniki | Thessaloniki, Greece | Charalampos Berberidis | 2417 |
| 3004 | Navy Hospital of Athens "NNA" | Athens, Greece | Gkikas Katsifis | 2117 |
| 4000 | Centro Medico del Angel S.C. | Mexicali, Mexico | Beatriz E. Zazueta | 13/983 |
| 4001 | Cemsi Policlinica | Culiacan, Mexico | Marco A.Maradiaga Cecena | 13/983 (2) |
| 4002 | Unidad de enfermedades reumaticas y cronico degenerativas | Torreon, Mexico | Isaura M.Rodriguez | 13/983 (1) |
| 5002 | Inland Rheumatolgoy Clinical Trials, INC | Upland, USA | Eric Lee | 28708/16 |
| 5003 | Altoona Center for Clinical Research | Duncansville, USA | Alan J. Kivitz | 28708/6 |
| 5004 | Low Country Rheumatology PA | North Charleston, USA | Clarence WLegerton III | 28708/17 |
| 5006 | West Tennessee Research Institute | Jackson, USA | Jacob A. Aelion | 28708/19 |
| 5014 | Arizona Arthritis & Rheumatology Research, PLLC | Phoenix, USA | Areena Swarup | 28708/5 |
| 5015 | Southwest Rheumatology | Mesquite, USA | Atul K Singhal | 28708/8 |
| 5016 | Summit Medical Group (New Jersey Physicians, LLC) | Clifton, USA | Marc A.Goldberg | 28708/10 |
| 5018 | Sun Valley Arthritis Center, Ltd | Peoria, USA | Joy Schechtman | 28708/14 |
| 5019 | IMC/Diagnostic & Medical Clinic | Mobile, USA | Daren Scroggie | 28708/1 |
| 5021 | Health Research of Oklahoma, PLLC | Oklahoma City, USA | Christine Codding | 28708/11 |
| 5022 | The Center for Rheumatology | Albany, USA | Joel M.Kremer | 28708/18 |
| 6000 | Hospital Garcia de Orta EPE | Almada, Portugal | Maria Jose Santos | FO / OF / 2014 / 2291 |
| 6001 | Instituto Portugues de Reumatologia | Lisboa, Portugal | Helena Santos | RS / OF / 2014 / 1773 |
| 6003 | Centro Hospitalar de Lisboa Ocidental Hospital Egas Moniz | Lisboa, Portugal | Fernando Pimentel Santos | RS / OF / 2014 / 1774 |
| 6005 | Centro Hospitalar de Lisboa Norte Hospital de Santa Maria | Lisboa, Portugal | Elsa Sousa | FO / OF / 2014 / 2211 |
| 7000 | Medical complex "Vashe Zdorovie" | Kazan, Russia | Svetlana P. Yakupova | #2 from 17 Feb 2014 |
| 7001 | St. Petersburg State Medical University named after Pavlov | St-Petersburg, Russia | Valery N.Marchenko | #159 from 27 Jan 2014 |
| 7002 | City Rheumatologic Hospital no.25 | St-Petersburg, Russia | Marianna S. Petrova | #12 from 09 Apr 2014 |
| 7003 | Rheumatology Inst of Russian Academy of Medical Sciences | Moscow, Russia | Marina L. Stanislav | #6 from 27 Feb 2014 |
| 7004 | City Rheumatologic Hospital no.25 | St Petersburg, Russia | Eugeny G.Zotkin | #9 from 19 Mar 2014 |
| 7051 | Hospital La Paz | Madrid, Spain | Alejandro Balsa | 2013/071 |
| 7052 | Hospital Universitario Marques de Valdecilla | Santander, Spain | Ricardo Blanco Alonso | 2013/071 |
| 7053 | Hospital Reina Sofia de Cordoba | Cordoba, Spain | Eduardo Collantes | 2013/071 |
| 7055 | Complejo Uni Hosp A Coruna antes Hospital Juan Canalejo | La Coruna, Spain | Francisco Javier Blanco | 2013/071 |
| 7057 | Corporacio Sanitaria Parc Tauli Sabadell | Sabadell, Spain | Jordi Gratacos | 2013/071 |
| 9001 | Whipps Cross Hospital | London, UK | Hasan I. Tahir | 14/EM/0086 |
| 9002 | Norfolk & Norwich University Hospital | Norwich, UK | Karl Gaffney | 14/EM/0086 |
| 9003 | Torbay Hospital | Torquay, UK | Kirsten Mackay | 14/EM/0086 |
